# Expert intuition vs. Algorithmic clustering: Mapping radiological subtypes in pulmonary sarcoidosis

**DOI:** 10.1371/journal.pone.0358960

**Published:** 2026-10-15

**Authors:** Justyna Sobczak, Adam J. Białas, Joanna Miłkowska-Dymanowska, Łukasz Gwadera, Patryk Sobczak, Katarzyna Torbiarczyk, Piotr Łacwik, Wojciech J. Piotrowski

**Affiliations:** Department of Pneumology, Medical University of Lodz, Lodz, Poland; Rutgers Biomedical and Health Sciences, UNITED STATES OF AMERICA

## Abstract

**Background:**

Pulmonary sarcoidosis is a heterogeneous granulomatous disease with a broad spectrum of clinical and radiological manifestations. While the traditional Scadding classification based on chest radiography remains a cornerstone of disease staging, it provides limited information regarding parenchymal involvement and poorly correlates with functional impairment. High-resolution computed tomography (HRCT) offers superior sensitivity in detecting subtle and early interstitial changes, yet standardized HRCT-based classification systems for sarcoidosis remain underdeveloped.

**Aim:**

This study aimed to propose and validate a novel HRCT-based classification of pulmonary sarcoidosis grounded in expert consensus, to assess its relationship with clinical symptoms, pulmonary function, and quality of life, and to compare it with data-driven radiological groupings obtained through cluster analysis.

**Methods:**

The study population included patients with confirmed pulmonary sarcoidosis managed at a tertiary referral center, either as inpatients or outpatients. HRCT scans were evaluated using a modified version of the previously published questionnaire, capturing 22 radiological features per lung lobe. Based on characteristic imaging patterns, four expert-defined subtypes were proposed: (1) Lymphonodal, (2) Disseminated-Nodular, (3) Central, and (4) Fibrotic. Symptom burden was assessed using the Fatigue Assessment Scale (FAS) and the Sarcoidosis Health Questionnaire (SHQ). Spirometric and gas transfer parameters (FEV_1_, FVC, T_L,CO_) were recorded. Statistical analyses included ANOVA, Spearman’s correlation, and hierarchical clustering (Ward’s method with Euclidean distances), with Cohen’s kappa used to evaluate concordance between classification systems.

**Results:**

Four distinct HRCT subtypes were identified through expert assessment, each reflecting varying degrees and distributions of parenchymal involvement. These subtypes differed significantly in clinical and functional presentation. The fibrotic subtype demonstrated the most severe functional impairment, with the highest prevalence of abnormal spirometry and diffusion deficits. In contrast, the lymphonodal subtype exhibited preserved lung function and minimal symptomatology. The Van den Heuvel radiological severity score showed strong correlation with radiological subtype and was significantly associated with FEV_1_, FVC, T_L,CO_, and SHQ scores. Unsupervised cluster analysis independently identified four data-driven radiological groups that partially overlapped with expert-defined subtypes, particularly in nodular and central patterns. However, the fibrotic subtype showed less distinct clustering, underscoring its radiological complexity. The agreement between expert-defined and cluster-derived classifications was limited (κ = 0.271), suggesting that the two approaches capture complementary but distinct dimensions of disease morphology.

**Conclusions:**

HRCT-based radiological subtypes of pulmonary sarcoidosis reflect clinically meaningful differences in symptom burden, ventilatory function, and radiological severity. While expert-driven and data-driven classifications show limited concordance, both provide valuable insights into disease heterogeneity. Radiological phenotyping using HRCT may serve as a practical adjunct to traditional staging systems, offering improved risk stratification and guiding personalized treatment decisions. Further prospective validation is needed to determine its utility in routine clinical practice and to explore its integration with emerging clinical and molecular phenotyping strategies.

## Introduction

Sarcoidosis is a multisystem granulomatous disorder characterized by the formation of non-caseating granulomas at sites of active inflammation. Although theoretically any organ system may be affected, pulmonary involvement is most common, occurring in over 90% of patients and typically presenting with bilateral hilar lymphadenopathy and interstitial lung abnormalities [1–6]. Given its frequency and clinical significance, thoracic imaging plays a central role in both the diagnosis and monitoring of sarcoidosis [7–9].

Chest radiography remains the primary imaging modality due to its accessibility, low cost, and limited radiation exposure. Scadding’s radiographic staging system, introduced in 1961, remains widely used in clinical practice and stratifies disease into four stages based on the presence and distribution of lymphadenopathy and parenchymal infiltrates [10]. While valuable, this system has limitations—including interobserver variability and poor correlation with functional parameters, particularly in intermediate stages [11–14]. Conventional radiography is also insensitive to early or subtle parenchymal changes, which may delay recognition of disease progression.

In selected clinical scenarios, such as atypical presentations, suspected comorbidities (e.g., infection, malignancy), or unexplained respiratory symptoms, computed tomography (CT), and more specifically high-resolution CT (HRCT), is employed for its superior sensitivity in detecting interstitial abnormalities [15,16]. HRCT has become a cornerstone in the evaluation of diffuse interstitial lung diseases, offering enhanced spatial resolution through reduced slice thickness and high-spatial-frequency reconstruction algorithms [17–22]. Beyond diagnosis, HRCT may also hold prognostic value. Radiological indices such as the main pulmonary artery to ascending aorta diameter ratio (MPAD/AAD) and the Composite Physiological Index (CPI) have been proposed to stratify risk and guide management in patients with interstitial lung disease, including sarcoidosis [23,24].

To enhance reproducibility and clinical interpretation of HRCT findings in sarcoidosis, Van den Heuvel developed a structured scoring system that captures a broad range of radiological abnormalities [25]. Recent reviews and international expert consensus further underscore the potential of CT to contribute to disease phenotyping, staging, and prognostication, particularly in complex or progressive cases [26,27].

This study aimed to identify and characterize the most prevalent HRCT-based radiological subtypes of pulmonary sarcoidosis, using a modified version of Van den Heuvel’s questionnaire. We also sought to determine whether these subtypes are associated with differences in clinical presentation, ventilatory impairment, and quality of life. Additionally, we compared this expert-driven classification to a data-driven approach using cluster analysis to assess the level of concordance and explore their complementary value in refining disease phenotyping.

## Materials and methods

### Study population and design

This cross-sectional study included patients with confirmed pulmonary sarcoidosis who were either hospitalized at the Department of Pneumology of the Medical University of Lodz, or managed on an outpatient basis at the affiliated Pulmonary Outpatient Clinic. Data from medical records were assessed from March 12, 2019, to September 1, 2023.

The study focused on identifying radiological subtypes of sarcoidosis using HRCT and correlating these with clinical, functional, and quality-of-life data. Radiological subtypes were defined by expert consensus based on characteristic HRCT patterns and included: Subtype 1 – Lymphonodal: Predominant hilar/mediastinal lymphadenopathy with ≤10% parenchymal involvement; Subtype 2 – Disseminated-Nodular: Widespread pulmonary nodules (>10% of lung surface), no fibrosis; Subtype 3 – Central: Perihilar and peribronchial confluent opacities; Subtype 4 – Fibrotic: Marked architectural distortion with bronchiectasis or honeycombing.

### Ethics

The Ethics Committee of Medical University of Lodz approved the study protocol (consent No RNN/87/19/KE from 12th March 2019). Patients’ approval was waived due to retrospective analysis of anonymised data.

### Radiological assessment

A modified version of the questionnaire published by Van den Heuvel et al. [25] was used to score HRCT images for 22 distinct radiological features. Each feature was assessed dichotomously (presence/absence) or semi-quantitatively (0–4 scale, based on the percentage of affected lobe surface). Individual lobes were assessed separately, and the total score reflected overall radiological severity.

Several modifications were made to align the tool with the available imaging: air trapping was excluded due to the lack of expiratory-phase scans; lymph node measurements were omitted due to the non-contrast nature of HRCT; predominant nodule size was documented in millimeters but excluded from the severity score.

Two experienced pulmonologists (over 30 and 10 years of ILD diagnostic experience) independently assessed the scans. Discrepancies were resolved by consensus.

### Clinical and functional assessment

Clinical symptoms, including fatigue, dyspnea, and cough, were evaluated using the Fatigue Assessment Scale (FAS) [28] and the Sarcoidosis Health Questionnaire (SHQ) [29], which incorporates both presence and severity ratings on a 7-point Likert scale.

Pulmonary function was assessed using standard spirometry and the transfer factor for carbon monoxide corrected for hemoglobin concentration (T_L,CO_).

Patients were divided into normal spirometry group and abnormal spirometry group: including obstructive, restrictive (suspected), nonspecific, or mixed patterns. Body plethysmography was not available for all patients to confirm restriction. Transfer factor for carbon monoxide was considered abnormal if below the 5th percentile of predicted T_L,CO_ values.

### Statistical analysis

Statistical analysis was performed using both descriptive and inferential methods to evaluate the relationship between radiological subtypes and clinical, functional, and radiological variables. Descriptive statistics included measures of central tendency and dispersion—mean, standard deviation, median, minimum, and maximum—for quantitative variables, and frequencies with corresponding percentages for qualitative variables.

For comparisons involving qualitative data, Fisher’s exact one-sided test was used for binary variables, while Pearson’s Chi-squared test was applied to non-binary categorical variables. Quantitative variables were first tested for normality of distribution. In cases where normality and homogeneity of variances were confirmed (Levene’s test p > 0.05), the Student’s t-test for independent samples was used. For non-normally distributed or semi-quantitative variables, the Mann–Whitney U test was applied. One-sided p-values were used in comparisons of quantitative and semi-quantitative variables.

Correlation analysis involving qualitative and semi-quantitative variables was conducted using Spearman’s rho with two-sided p-values. To assess differences in continuous variables across multiple radiological subtypes, one-way analysis of variance (ANOVA) was used. When the assumption of equal variances was met (Levene’s test p > 0.05), post-hoc comparisons were performed using Tukey’s HSD test. In cases of unequal variances (Levene’s test p < 0.05), the Games–Howell test was applied, and the Welch-corrected F-statistic was used to determine significance.

ANOVA models were constructed in a stepwise manner, starting with single dependent variables selected based on their strength of correlation with the radiological subtypes. To avoid multicollinearity, only uncorrelated variables were included in multifactorial models. All variables were standardized prior to inclusion, and sigma transformation was applied to categorical variables.

For unsupervised classification, both agglomerative hierarchical clustering using Ward’s method and Euclidean distances, and the k-means clustering method were employed. To evaluate the concordance between expert-defined subtypes and data-driven clusters, Cohen’s kappa statistic was calculated. Additionally, Spearman’s correlation and Pearson’s Chi-squared test were used to examine the distribution of individual radiological features across both classification schemes.

## Results

### Radiological subtypes

Based on detailed analysis of high-resolution computed tomography (HRCT) images and the clinical expertise of physicians specialized in interstitial lung diseases, four predominant radiological subtypes of pulmonary sarcoidosis were proposed. These subtypes represent the most frequently observed patterns in the study cohort and were developed to enhance radiological classification beyond traditional staging, with potential implications for prognosis and treatment planning and were summarized in Tables 1 and 2.

**Table 1:** Radiological subtypes description.

Subtype		Description
Subtype 1	**Lymphonodal**	This subtype is defined by predominant bilateral hilar and mediastinal lymphadenopathy, with minimal parenchymal involvement (≤10% of total lung surface area). Radiological features consistent with pulmonary fibrosis or the presence of aspergilloma constitute exclusion criteria for this subtype (Fig 1).
Subtype 2	**Disseminated-nodular**	This pattern is characterized by numerous, diffusely scattered pulmonary nodules (typically <5 mm in diameter), distributed throughout the lung parenchyma and independent of nodal involvement. Lung involvement exceeds 10% of the surface area. As with Subtype 1, the presence of fibrosis or aspergilloma excludes classification under this subtype (Fig 2).
Subtype 3	**Central**	This subtype presents as extensive, confluent perihilar opacities with radiating extension toward the peripheral lung fields, often encasing the central bronchi. It reflects significant central airway involvement and may resemble a mass-like distribution pattern (Fig 3).
Subtype 4	**Fibrotic**	This advanced radiological phenotype is defined by marked architectural distortion of the lung parenchyma, including traction bronchiectasis, bronchial deformity, and honeycombing. The presence of aspergilloma or other signs of irreversible pulmonary damage are also indicative of this subtype (Fig 4).

*Materials from the collections of the Department of Pneumology, Medical University of Lodz.

**Fig 1:**
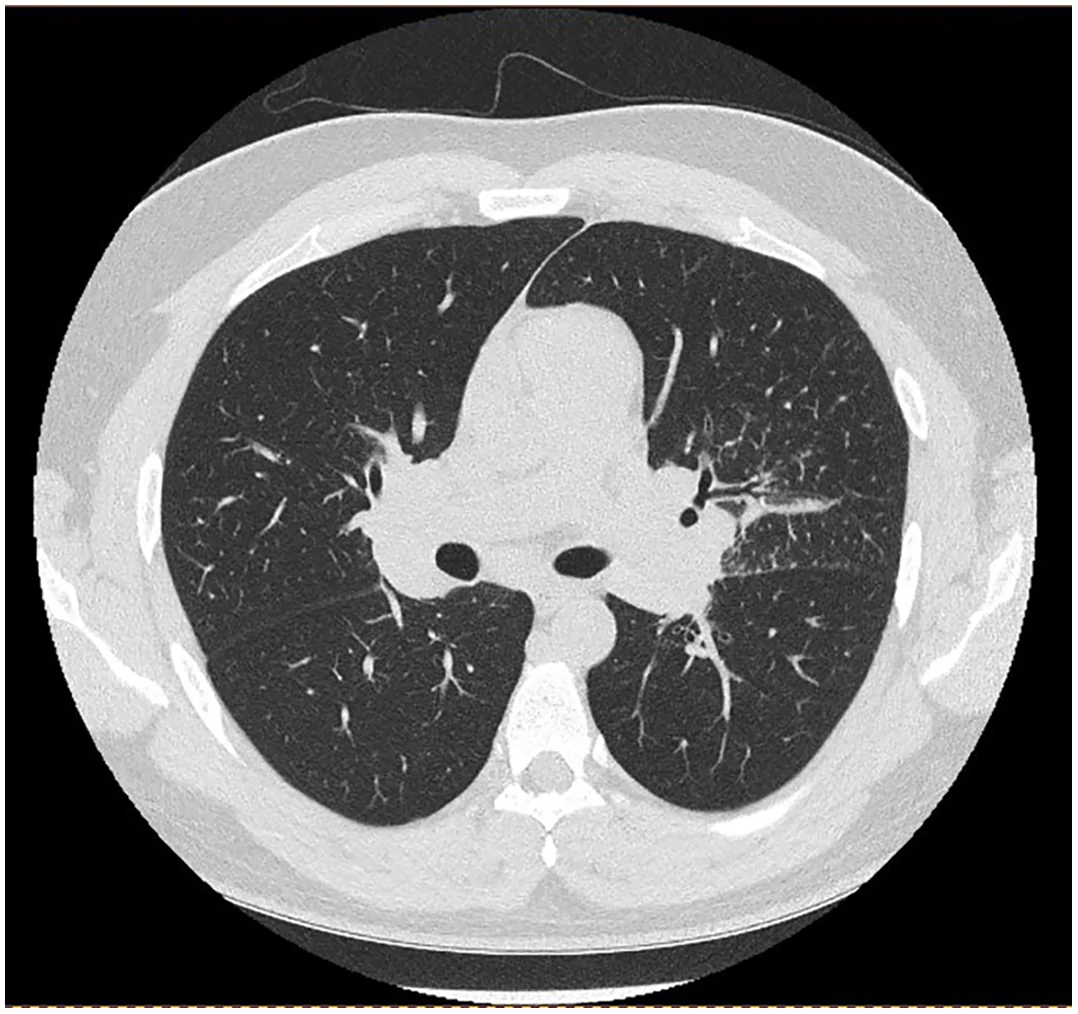
Representative HRCT image of subtype 1 – Lymphonodal. A 33-year-old male with pulmonary sarcoidosis. The scan demonstrates prominent bilateral hilar lymphadenopathy with minimal parenchymal involvement. Subtle, scattered peribronchial nodules are visible, occupying less than 10% of the total lung surface area.

**Fig 2:**
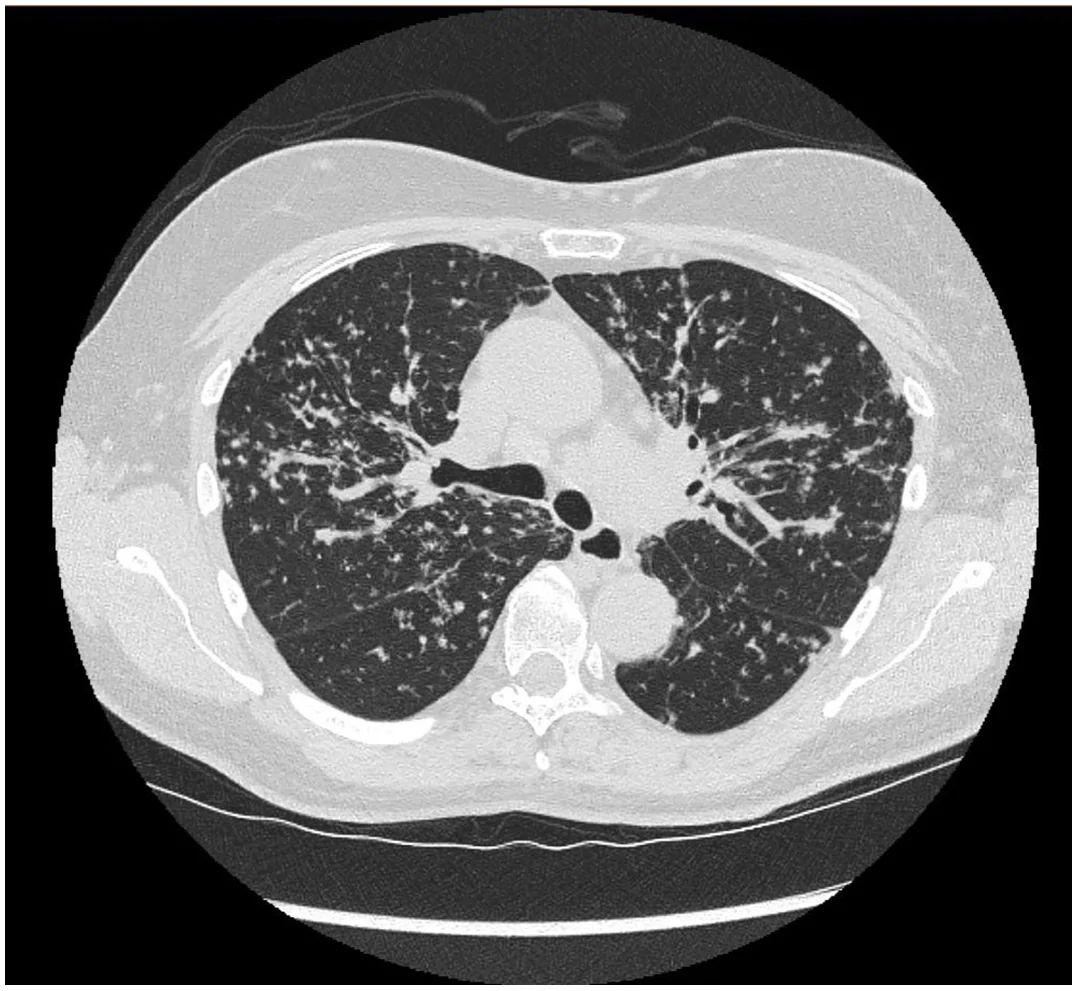
Representative HRCT image of Subtype 2 – Disseminated-nodular. A 62-year-old female with pulmonary sarcoidosis. The scan reveals numerous small nodules with a predominant peribronchial distribution, extensively involving the middle and upper lung lobes.

**Fig 3:**
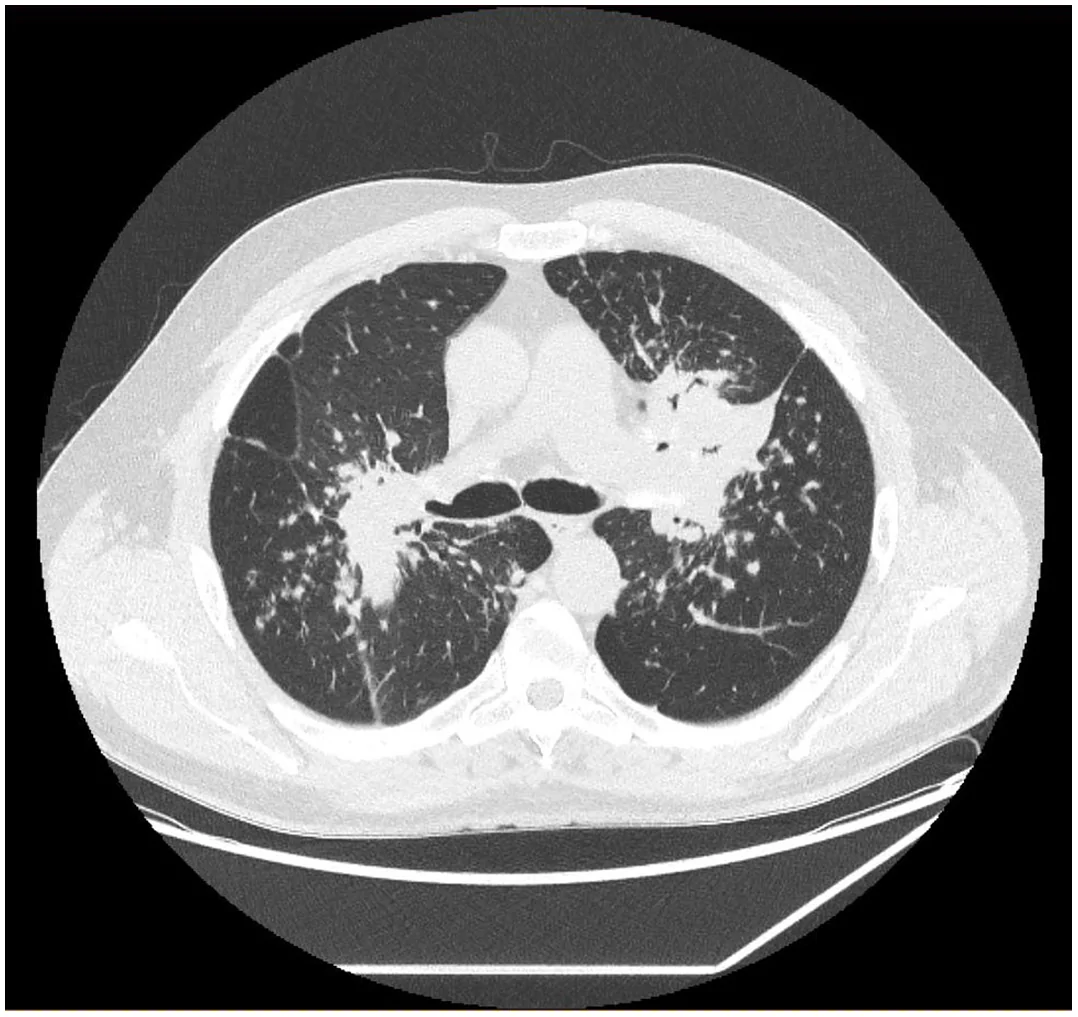
Representative HRCT image of Subtype 3 – Central. A 63-year-old male with pulmonary sarcoidosis. The HRCT reveals nodular opacities predominantly distributed in the perihilar regions, accompanied by central peribronchial consolidations.

**Fig 4:**
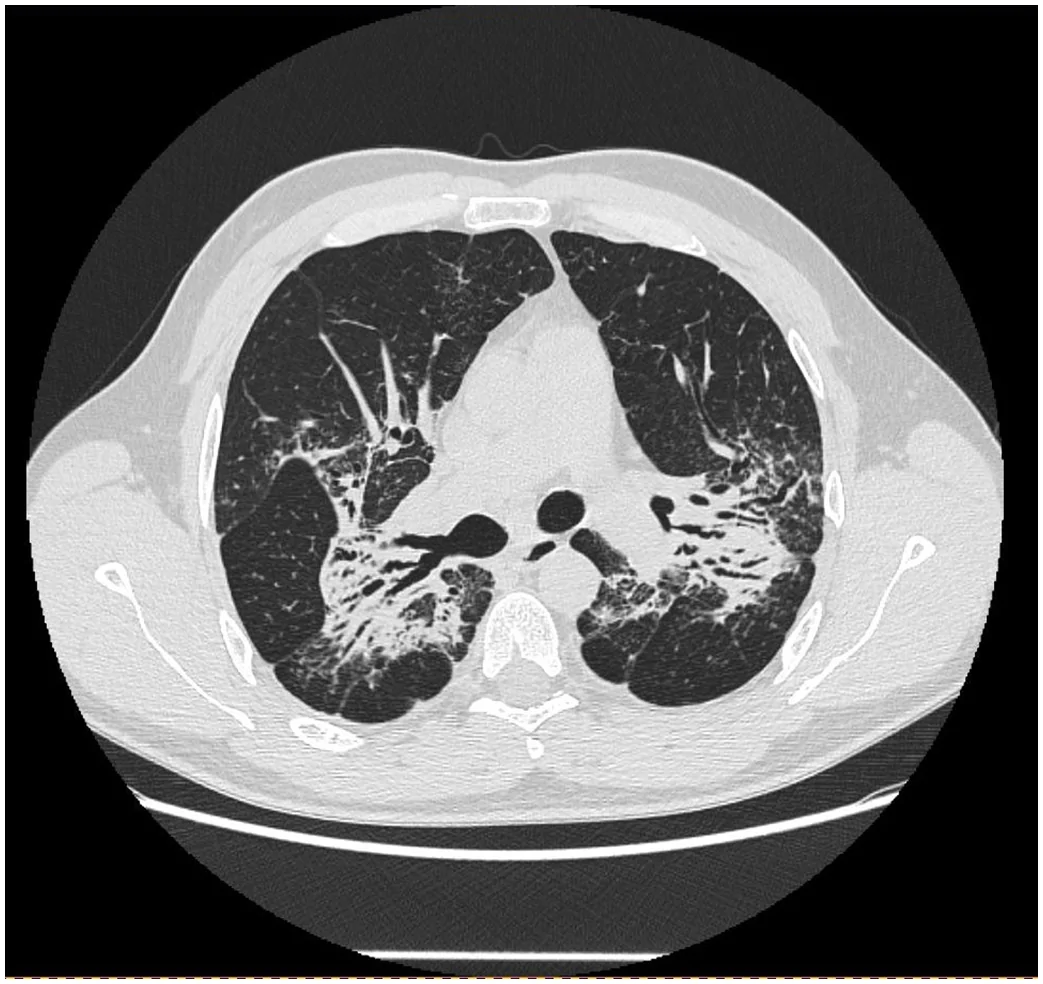
Representative HRCT image of Subtype 4 – Fibrotic. A 36-year-old male with pulmonary sarcoidosis. The HRCT scan demonstrates features of advanced pulmonary fibrosis, including marked architectural distortion and the presence of traction bronchiectasis.

**Table 2:** Prevalence of radiological subtypes in the study cohort.

Radiological Subtype	Number of Occurrences	Percentage of Occurrences
1	61	54,96%
2	31	27,93%
3	14	12,61%
4	5	4,50%

Radiological subtypes were characterized based on specific imaging features assessed using a modified version of Van den Heuvel’s questionnaire. For subtype classification, each radiological feature was evaluated dichotomously—recorded solely by its presence or absence—irrespective of the anatomical distribution or extent across individual lung lobes. Variables such as the predominant nodule size and the percentage of affected lobe area were excluded from this analysis due to their qualitative and descriptive nature and are not included in the summary table (Figs 1, 2, 3, 4).

Associations between individual radiological subtypes and specific imaging features were evaluated, with detailed results presented in **S1** **Table** **(Supporting Information Tables)** (Table 3).

**Table 3:** Associations between individual radiological subtypes and specific imaging features.

Subtype	Correlation
**Subtype 1**	demonstrated a positive correlation exclusively with hilar and mediastinal lymphadenopathy. Negative correlations were observed with most other features, with the exception of ground-glass, mosaic pattern, and parenchymal calcifications, for which no statistically significant relationship was established
**Subtype 2**	was associated with nearly half of the assessed features. Positive correlations were identified for bronchovascular, parenchymal, subpleural, and sharp-marginated nodules, as well as architectural distortion, septal lines, bronchial deformation, and bronchiectasis. Conversely, a negative correlation was found with hilar and mediastinal lymphadenopathy, distinguishing this subtype from Subtype 1
**Subtype 3**	exhibited positive correlations with the majority of evaluated radiological features, excluding ground-glass, mosaic pattern, bronchiectasis, pleural thickening, and parenchymal calcifications, with which no significant associations were observed
**Subtype 4**	was positively correlated with central consolidations, architectural distortion, bronchial deformations, bronchiectasis, and interstitial calcifications, reflecting the advanced and destructive nature of this radiological phenotype

Notably, **no statistically significant correlations** were found between any of the subtypes and the presence of **ground-glass or mosaic pattern**, indicating that these features may not reliably distinguish between radiological phenotypes of sarcoidosis. Moreover, **aspergilloma** was not identified in the study cohort, precluding its analysis.

Furthermore, the frequency of individual radiological features was compared across the four proposed subtypes, with results presented in **S2** **Table** **(Supporting Information Tables)**. Statistically significant differences were observed among the subtypes for nearly all features, except for **ground-glass**, **mosaic pattern**, and **parenchymal calcifications**, which did not significantly differ across groups.

Enlargement of hilar and mediastinal lymph nodes was a common finding across all subtypes, though most prominent in Subtypes 1 and 3 (100% in both), and somewhat less frequent in Subtypes 2 (80.65%) and 4 (80%). Subtype 1 demonstrated only minimal interstitial abnormalities, consistent with its classification criteria. In contrast, Subtypes 2, 3, and 4 showed a higher and comparable prevalence of interstitial changes, particularly: bronchovascular nodules were observed in 22.03% (Subtype 1), 67.74% (Subtype 2), 100% (Subtype 3), and 20.00% (Subtype 4); parenchymal nodules were found in 30.51% (Subtype 1), 100% (Subtype 2), 100% (Subtype 3), and 80.00% (Subtype 4); subpleural nodules occurred in 55.93% (Subtype 1), 96.77% (Subtype 2), 100% (Subtype 3), and 100.00% (Subtype 4).

The distribution of central consolidations further supported the distinction of Subtype 3, where this feature was present in 85.71% of patients, compared to 5.08% in Subtype 1, 19.45% in Subtype 2, and 60.00% in Subtype 4.

Architectural distortion, a marker of parenchymal remodeling and disease chronicity, was identified in 16.95% (Subtype 1), 54.8% (Subtype 2), 71.43% (Subtype 3), and 80.00% (Subtype 4). Similarly, bronchial deformation followed a comparable trend: 8.47% (Subtype 1), 61.29% (Subtype 2), 78.57% (Subtype 3), and 80.00% (Subtype 4).

Bronchiectasis was most prominent in the fibrotic subtype (60.00%), but also present in 8.47% (Subtype 1), 41.94% (Subtype 2), and 14.29% (Subtype 3), reflecting varying degrees of airway involvement.

The presence of aspergilloma could not be analyzed due to the absence of confirmed cases within the study population.

These findings reinforce the distinct radiological patterns associated with each subtype and support their relevance in differentiating disease phenotype and potentially guiding clinical management.

### Cluster analysis and identification of radiological groups

To complement the expert-defined radiological subtypes, an unsupervised cluster analysis was conducted based on the presence or absence of radiological features. Using Ward’s method with Euclidean distances, four distinct clusters of imaging patterns were identified. These data-driven clusters are referred to as **Groups A, B, C,** and **D**.

Among the identified clusters, **Group A** demonstrated the greatest dissimilarity compared to the other groups, indicating a unique combination of radiological features. In contrast, **Groups B and C** were the most closely related, sharing similar imaging characteristics. The relationships and proximities between these clusters are visualized in Fig 5, which illustrates the dendrogram resulting from the hierarchical clustering process.

**Fig 5:**
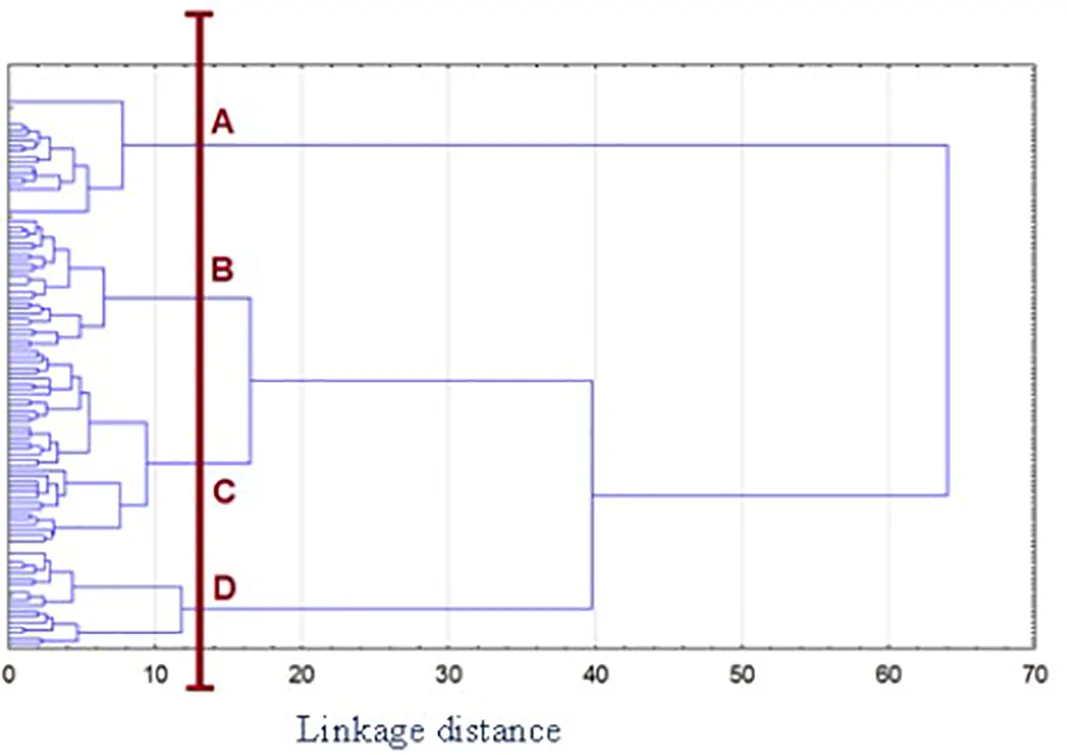
Cluster analysis of radiological features in individual patients with group differentiation.

The chart presents the outcome of hierarchical cluster analysis, where radiological features were treated as binary variables progressively aggregated into increasingly broader clusters. The horizontal axis of the dendrogram represents **agglomerative distances** (Euclidean), indicating the relative dissimilarity between merged elements.

Each **node** on the diagram marks the agglomerative distance at which two elements or clusters were combined. As the clustering progresses, individual patients with similar radiological patterns are grouped, ultimately resulting in a comprehensive clustering of all observations.

The **red horizontal line** represents the cutoff point used to define **four distinct clusters**, labeled from top to bottom as **Groups A, B, C,** and **D**. This threshold was selected to balance within-group similarity and between-group distinctiveness.

The diagram clearly demonstrates that **Group A** exhibits the highest degree of dissimilarity relative to the other clusters, suggesting a distinct radiological phenotype. In contrast, **Groups B and C** are the most closely related, reflecting similar distributions of radiological features. This clustering provides a complementary, data-driven classification that can be compared with the expert-defined subtypes for validation and refinement of radiological profiling in sarcoidosis.

This approach provides an objective, feature-based classification of radiological phenotypes and offers a framework for evaluating the agreement and complementarity between expert-driven subtyping and statistical clustering methods (Table 4).

**Table 4:** Prevalence of individual radiological groups.

Radiological group	Number of Occurrences	Percentage of Occurrences
A	20	18,02%
B	28	25,23%
C	43	38,74%
D	20	18,02%

A comparative analysis of the frequency of individual radiological features across the four data-driven clusters (Groups A–D) was performed and is summarized in S3 Table **(Supporting Information Tables).** Statistically significant differences were identified among the groups for nearly all features, with the exception of ground-glass and mosaic pattern, which showed no significant variation across clusters.

Hilar and mediastinal lymphadenopathy was present in all groups, with prevalence ranging from 79.17% in Group A to 100% in Groups B and D, and 94.44% in Group C. Despite universal presence, the frequency varied, reflecting different levels of nodal involvement.

Distinct patterns of parenchymal involvement were evident across the clusters: bronchovascular nodules: Group A – 54.17%, Group B – 0%, Group C – 100%, Group D – 20.00%; parenchymal nodules: Group A – 91.67%, Group B – 7.14%, Group C – 94.44%, Group D – 36.84%; subpleural nodules: Group A – 87.50%, Group B – 17.86%, Group C – 97.22%, Group D – 100.00%

Central consolidations were most frequently observed in Group C (55.56%), with markedly lower frequencies in Group A (8.33%), Group B (3.57%), and Group D (5.26%).

Markers of architectural disruption were most pronounced in Group C, consistent with a more advanced or fibrotic phenotype: architectural distortion: Group A – 29.17%, Group B – 14.29%, Group C – 75.00%, Group D – 15.79%; bronchial deformation: Group A – 25.00%, Group B – 3.57%, Group C – 75.00%, Group D – 21.05%; bronchiectasis: Group A – 25.00%, Group B – 3.57%, Group C – 41.67%, Group D – 5.26%

The presence of aspergilloma could not be assessed due to the absence of such cases within the study cohort.

These findings support the distinctiveness of the identified radiological clusters and underscore the potential clinical relevance of this data-driven classification in stratifying patients by disease pattern and severity.

### Comparison of radiological subtypes and radiological groups

Correlation analysis revealed a nonlinear relationship between the statistically derived radiological groups (A–D) and the expert-defined radiological subtypes 1 to 4, as summarized in Table 5. While some degree of alignment was observed, the correspondence was not uniformly strong across all subtypes. Group B demonstrated a positive correlation with Subtype 1 (Lymphonodal), reflecting the predominance of isolated hilar and mediastinal lymphadenopathy with minimal parenchymal involvement in both classifications. Group A correlated positively with Subtype 2 (Disseminated-nodular), consistent with the shared presence of widespread nodular involvement without fibrotic features. Group C was positively associated with Subtype 3 (Central), characterized by extensive perihilar and peribronchial consolidations.

**Table 5:** Correlation analysis between radiologic subtypes and radiologic groups.

	Group A	Group B	Group C	Group D
Subtype 1	−0,1251	**0,5576**	**−0,6413**	**0,2881**
Subtype 2	**0,2493**	**−0,3802**	**0,2429**	−0,1350
Subtype 3	−0,1422	**−0,2310**	**0,4862**	−0,1803
Subtype 4	−0,0129	−0,1318	**0,2172**	−0,1029

*Statistically significant differences are highlighted in bold (p < 0.05).

In contrast, Subtype 4 (Fibrotic) did not show a strong correlation with any of the statistically derived groups.

Additionally, the kappa test revealed weak agreement between the radiologic groups and radiologic subtypes (κ = 0.271).

### Analysis of differences between radiologic subtypes

#### • Assessment of the relationship between radiological subtype and clinical parameters, symptoms, and demographic factors.

Based on the CT scan, the radiological subtype was determined for each patient. An analysis was then performed to evaluate associations between the radiological subtypes and the previously described clinical parameters, sarcoidosis-related symptoms, and demographic data.

No significant differences in the distribution of radiological subtypes were observed between age groups (<35 vs. ≥ 35 years; *p* > 0.05) or between sexes (*p* > 0.05).

The radiological subtype showed a strong positive correlation with the Van den Heuvel questionnaire score (*R* = 0.776, *p* < 0.001), and significant negative correlations with FEV_1_ (*R* = −0.447, *p* < 0.001), FVC (*R* = −0.330, *p* = 0.001), and T_L,CO_ (*R* = −0.307, *p* = 0.002). Positive correlations were also found with abnormal diffusion capacity (*R* = 0.335, *p* = 0.001) and abnormal spirometry (*R* = 0.270, *p* = 0.006). No other statistically significant correlations were identified (*p* > 0.05).

Significant differences were observed in the prevalence of abnormal spirometry results across radiological subtypes (*p* = 0.0017), with the highest frequency in Subtype 4 (80.00%) and the lowest in Subtypes 1 and 2 (17.86% and 17.24%, respectively).

Differences in the frequency of abnormal diffusion capacity were also significant (*p* = 0.0008), being most common in Subtype 4 (90.57%) and least frequent in Subtype 1 (9.43%).

A series of statistical tests conducted during the development of the one-way ANOVA model demonstrated that the **Van den Heuvel score** exhibited the strongest association with radiological subtype (*η^2^* = 0.62, *p* < 0.001), indicating a substantial proportion of variance explained by this variable.

Table 6 presents the results of **pairwise comparisons** of the mean Van den Heuvel scores across the four radiological subtypes 1 to 4. Statistically significant differences were identified between Subtype 1 and each of Subtypes 2, 3, and 4 (*p* < 0.001), as well as between Subtypes 2 and 3 (*p* = 0.020), highlighting the discriminatory capacity of this score in reflecting radiological severity.

**Table 6:** Pairwise comparisons of mean Van den Heuvel scores by radiological subtype.(*Values to be inserted based on dataset output*).

	Subtype 1	Subtype 2	Subtype 3	Subtype 4
Subtype 1		**<0,001**	**<0,001**	**<0,001**
Subtype 2	**<0,001**		**0,020**	0,053
Subtype 3	**<0,001**	**0,020**		0,941
Subtype 4	**<0,001**	0,053	0,941	

The presented results display pairwise comparisons of mean Van den Heuvel scores across radiological subtypes [1–4]. The values in the table represent *p*-values indicating the statistical significance of differences in mean scores between each pair of subtypes. Entries along the diagonal (from the top-left to the bottom-right corner) represent comparisons within the same subtype and are therefore omitted.

### Dendrogram/ Ward’s Method/ Euclidean distance

Significant differences were identified between **radiological subtype 1** and **subtypes 2, 3,** and **4** (*p* < 0.001), reflecting a significantly lower radiological burden in subtype 1. Additionally, a significant difference was observed between **subtypes 2 and 3** (*p* = 0.020), suggesting a progressive increase in radiological severity from subtype 2 to –3.

All one-way ANOVA analyses are presented in S4 Table **(Supporting Information Tables).** Among the models tested, those incorporating **FEV**_**1**_, **abnormal diffusion capacity**, and **abnormal spirometry** significantly explained variation in radiological subtypes (*p* < 0.05, *η^2^* > 0.14), indicating a moderate to strong association between these functional parameters and the imaging-based classification.

For other clinical or demographic variables that did not demonstrate significant correlation with radiological subtype, the construction of a meaningful ANOVA model was not possible (*p* > 0.05).

Attempts to construct ANOVA models using combinations of two dependent variables resulted in a reduced explanatory capacity (*η²*) and were hindered by the presence of strong internal correlations between the analyzed variables. As a result, these multifactorial models were deemed statistically unstable and were not further pursued.

### Classification analysis

A hierarchical cluster analysis was performed using Ward’s method with Euclidean distances to assess the similarity between radiological subtypes. The analysis revealed that the shortest distance—indicating the greatest similarity—was between **subtypes 2 and 3** (1.75), followed by **subtypes 1 and 2** (1.80), **3 and 4** (2.62), **1 and 3** (3.20), **2 and 4** (4.22), and the largest distance between **subtypes 1 and 4** (5.55), reflecting the greatest dissimilarity. These results are illustrated in Fig 6.

**Fig 6:**
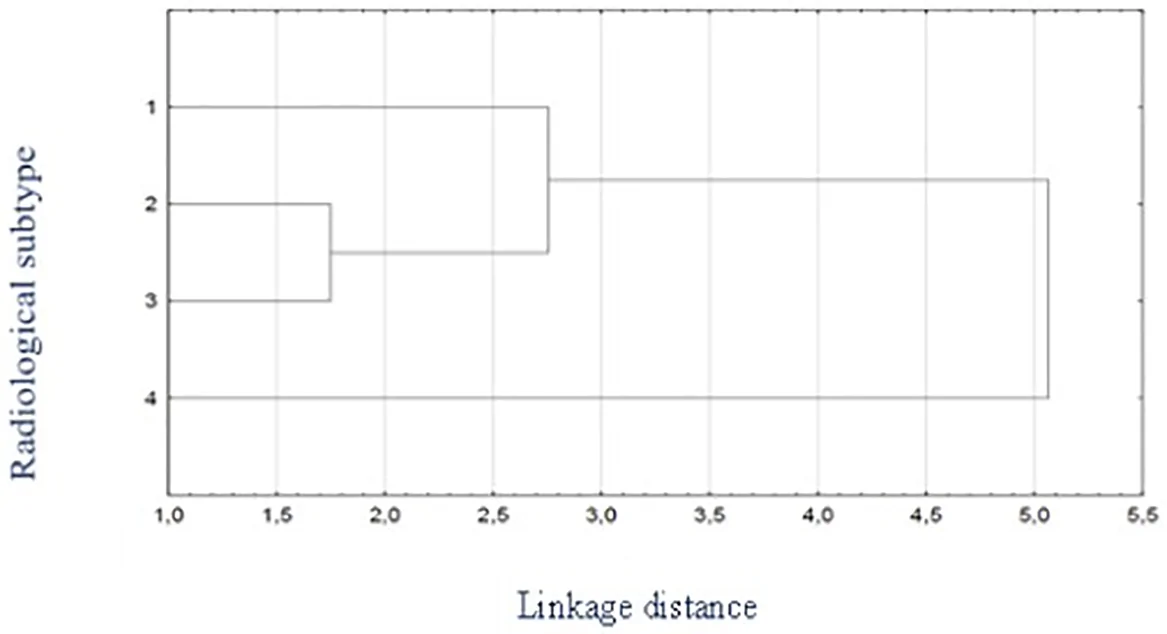
Euclidean distances in hierarchical classification using Ward’s method for radiological subtypes.

Dendrogram illustrating the hierarchical relationships between the four radiological subtypes based on Euclidean distances. Ward’s method was applied to cluster the subtypes according to their radiological feature profiles. The shortest distance was observed between **subtypes 2 and 3 (1.75)**, indicating the highest similarity, followed by **subtypes 1 and 2 (1.80)**. The greatest dissimilarity was observed between **subtypes 1 and 4 (5.55)**, suggesting distinct radiological patterns.

## Discussion

This study proposed and validated a novel radiological classification system for pulmonary sarcoidosis based on HRCT morphology, identifying four distinct subtypes: lymphonodal, disseminated-nodular, central, and fibrotic. These subtypes were derived through expert consensus and reflected specific patterns of lesion distribution and parenchymal involvement. Importantly, the fibrotic subtype was associated with the most severe structural changes, while the lymphonodal subtype showed minimal parenchymal abnormalities.

A key finding of this study is the significant association between radiological subtype and clinical manifestations. Patients with greater parenchymal involvement, particularly those with fibrotic or central subtypes, exhibited more pronounced ventilatory impairment and reported worse quality of life. In contrast, those with lymphonodal disease maintained preserved lung function and reported milder symptoms. These observations suggest that HRCT-based subtyping may offer a valuable prognostic tool and inform individualized treatment strategies, potentially supporting decisions regarding earlier therapeutic intervention in patients with more aggressive radiological patterns.

Previous classification systems, such as Scadding’s radiographic staging, have played a foundational role in disease assessment. Scadding demonstrated that spontaneous remission is most likely in stage I and becomes progressively less frequent with advancing stage, with stage IV being associated with irreversible fibrosis [10,11]. Muers et al. later proposed a classification based on the character and extent of radiographic opacities, establishing a link between radiological findings and functional decline over time in corticosteroid-treated patients [11,12].

Prasse and colleagues introduced the Sarcoid Clinical Activity Classification (SCAC), aligning clinical trajectories with radiological stages. Their findings confirmed the association between stage IV disease and chronic, treatment-refractory phenotypes, but also revealed that patients with intermediate radiological stages (II and III) showed highly variable clinical courses that defied consistent classification. These findings highlight the limitations of stage-based systems and support the rationale for a morphology-driven, CT-based classification that may capture disease behavior more accurately [13,14].

The superior diagnostic utility of HRCT over chest radiography has been well established. Mathieson et al. demonstrated significantly greater diagnostic confidence and accuracy with HRCT among experienced observers, reinforcing its role in the assessment of diffuse parenchymal lung disease [22]. Moreover, growing recognition of sarcoidosis as a heterogeneous spectrum of disorders, rather than a single entity, has led to efforts to define CT-based phenotypes. In a recent international Delphi study, consensus was achieved on seven distinct HRCT phenotypes of pulmonary sarcoidosis, categorized broadly into non-fibrotic and fibrotic subtypes [27]. These efforts, including the current study, contribute to a shift toward more precise, image-based phenotyping, with potential to enhance both prognostic evaluation and treatment decision-making.

In addition to expert-driven classification described in this study, a parallel unsupervised cluster analysis based on HRCT features identified four data-driven radiological groups. While there was partial concordance between the two approaches, especially in nodular and central phenotypes, the fibrotic subtype appeared less consistently captured through statistical clustering, highlighting its complex and variable presentation. The modest agreement between expert and algorithmic classification underscores the multifaceted nature of sarcoidosis and the potential need for integrated, multimodal phenotyping frameworks.

The question of which of these classifications holds greater value remains open. However, the classification based on clinical experience appears to offer an advantage in prognostic assessment.

Lin et al. used cluster analysis to objectively classify sarcoidosis based on clinical and radiological variables, identifying six prognostically relevant subgroups differing in disease severity and immunosuppressive treatment use [30,31]. In the context of the aforementioned considerations, integrating clinical expertise with mathematical and informatics methodologies appears to be a promising approach. A computer program, based on data selected by the clinician, could create a certain pattern (algorithm) and then use it for further analyses, including prognosis assessment.

Machine learning (ML) refers to the study of algorithms and statistical models that enable computer systems to perform specific tasks without the need for explicit instructions. Instead, these systems rely on patterns and inference to make decisions. ML algorithms construct a mathematical model based on sample “training” data to make predictions or decisions without requiring additional programming for task execution [32]. There are numerous applications of ML in the field of medicine, particularly in radiology. It has been demonstrated that the analysis of pulmonary nodules using low-dose computed tomography images serves as a valuable tool for predicting lung cancer [33]. The study published by Vasconselos et al. demonstrated the usefulness of machine learning (ML) in differentiating specific patterns of interstitial lung disease, thereby enhancing the accuracy of diagnosis [34].

Kim et al. selected a series of images depicting healthy lungs and various pathologies, including emphysema, cryptogenic organizing pneumonia, and acute interstitial pneumonia. Based on these images, the authors identified six distinct patterns: normal lung, ground-glass opacity, consolidation, reticular changes, emphysema, and honeycombing. The training set of patterns, which served as the reference point for machine learning, consisted of several hundred images, selected and defined by two experienced radiologists. The utility of ML was verified on a test set of images [35].

To date, sarcoidosis has not been the focus of comparable studies. Considering the presence of characteristic radiological features, alongside the remarkable variability of these features, this issue would provide a broad field for further research and discoveries. One of the goals could be to differentiate sarcoidosis from other interstitial lung diseases. In the context of this study, machine learning seems potentially useful in defining various clusters of radiological symptoms, linking them to specific disease courses and prognoses. However, this requires extensive research and collaboration among specialists from different medical fields involved in the care of patients with sarcoidosis.

In summary, the HRCT-based classification proposed in this study demonstrates strong correlations with clinical and functional measures of disease severity, offers more detailed stratification than traditional radiographic staging, and may serve as a useful adjunct in the personalized management of pulmonary sarcoidosis. Further prospective studies are warranted to validate its prognostic value and explore its integration with clinical and molecular phenotyping frameworks.

This study presents several limitations that should be considered when interpreting the findings. First, its cross-sectional design precludes any conclusions regarding the temporal evolution of radiological patterns or causality between radiological subtypes and clinical or functional parameters. Longitudinal studies would be required to assess the prognostic implications and progression trajectories of the identified subtypes. Second, the study was conducted in a single tertiary care center, potentially introducing selection bias toward more symptomatic or complex cases. Consequently, the distribution of radiological subtypes and clinical characteristics may not be fully representative of the broader sarcoidosis population, particularly those with milder or asymptomatic disease. Third, body plethysmography was not available for all patients, limiting the ability to definitively confirm restrictive ventilatory defects. As a result, some cases categorized under “suspected restriction” may have been misclassified, thereby affecting the accuracy of associations between radiological subtype and pulmonary function. Fourth, certain radiological features, such as air trapping and lymph node dimensions, were excluded from analysis due to the lack of expiratory-phase and contrast-enhanced HRCT imaging, respectively. This may have limited the comprehensiveness of the radiological assessment and the ability to detect subtle airway involvement or quantify nodal burden. Fifth, the expert-defined radiological subtypes were based on consensus interpretation by two experienced pulmonologists. While this enhances clinical relevance, it introduces a degree of subjectivity and potential observer bias. Although efforts were made to reach consensus, inter-observer variability was not formally quantified. Finally, the absence of histopathological correlation, particularly for fibrotic changes, and the exclusion of patients with aspergilloma (none identified in this cohort) limit the generalizability of the findings to patients with more advanced or complicated sarcoidosis phenotypes. Future studies incorporating longitudinal follow-up, multicenter cohorts, comprehensive pulmonary function testing, and histopathological validation are warranted to refine the proposed classification system and validate its clinical utility.

## Conclusions

HRCT enables the identification of distinct radiological subtypes of pulmonary sarcoidosis, whether through expert-driven clinical assessment or data-driven cluster analysis. While both methods capture meaningful patterns of disease, their limited concordance highlights the need for further investigation into their respective diagnostic and prognostic utility.

Importantly, the proposed HRCT-based subtypes correlate with key clinical parameters, including the severity of ventilatory impairment and symptom burden. These findings underscore the practical relevance of radiological phenotyping in informing disease assessment and guiding individualized management. Future studies are warranted to validate these subtypes prospectively and to determine how they may best complement or enhance existing staging systems in routine clinical practice.

## Supporting information

S1 Table The correlations between the different radiologic subtypes and selected features described in imaging studies. (DOCX)

S2 Table The frequency with which specific radiological features occurred in different radiological subtypes. (DOCX)

S3 Table Frequency of individual radiological features observed in different groups. (DOCX)

S4 Table One-way ANOVA models explaining the radiological subtype. (DOCX)
