# Supplementary material for: Expert intuition vs. Algorithmic clustering: Mapping radiological subtypes in pulmonary sarcoidosis
Source: PLoS One. 2026 Oct 15;21(10):e0358960. doi: 10.1371/journal.pone.0358960 (PMC13671569; doi:10.1371/journal.pone.0358960)
Supplement: Supplementary file 1 [file pone.0358960.s001.docx]

**Table S1 . The correlations between the different radiologic subtypes and selected features described in imaging studies**.

|  | Subtype 1 | Subtype 2 | Subtype 3 | Subtype 4 |
| --- | --- | --- | --- | --- |
| Hilar and mediastinal lymphadenopathy | **0,2846** | **-0,3326** | 0,1006 | -0,1214 |
| Lymph node calcifications | **-0,1969** | 0,0011 | **0,2624** | 0,0469 |
| Bronchovascular nodules | **-0,5005** | **0,2888** | **0,4248** | -0,1100 |
| Parenchymal nodules | **-0,6910** | **0,4991** | **0,3039** | 0,0835 |
| Subpleural nodules | **-0,4856** | **0,3146** | **0,2203** | 0,1258 |
| Confluent nodules | **-0,4981** | 0,1741 | **0,4751** | 0,0512 |
| Sharp-marginated nodules | **-0,4573** | **0,2243** | **0,3050** | 0,1177 |
| Peripheral consolidation | **-0,3197** | 0,0637 | **0,3116** | 0,1210 |
| Central consolidations | **-0,4439** | -0,0405 | **0,5901** | **0,2010** |
| Gound-glass | -0,0373 | 0,0020 | 0,0296 | 0,0371 |
| Mosaic pattern | 0,0818 | -0,0149 | -0,0749 | -0,0428 |
| Architectural distortion | **-0,4634** | **0,2242** | **0,2680** | **0,1919** |
| Septal line | **-0,6049** | **0,3874** | **0,3050** | 0,1177 |
| Honeycombing | **-0,3069** | 0,1227 | **0,3444** | -0,0842 |
| Bronchial deformation | **-0,6188** | **0,3355** | **0,3427** | **0,2023** |
| Bronchiectasis | **-0,3361** | **0,3219** | -0,0641 | **0,2090** |
| Pleural thickening | **-0,3151** | 0,1600 | 0,1648 | 0,1419 |
| Parenchymal calcifications | -0,1141 | -0,0149 | 0,0709 | **0,1904** |
| Aspergiloma | - | - | - | - |

*Statistically significant correlations (p < 0.05) are marked in bold.
