# Supplementary material for: Expert intuition vs. Algorithmic clustering: Mapping radiological subtypes in pulmonary sarcoidosis
Source: PLoS One. 2026 Oct 15;21(10):e0358960. doi: 10.1371/journal.pone.0358960 (PMC13671569; doi:10.1371/journal.pone.0358960)
Supplement: Supplementary file 2 [file pone.0358960.s002.docx]

**Table S2. The frequency with which specific radiological features occurred in different radiological subtypes.**

|  | Subtype 1 | Subtype 2 | Subtype 3 | Subtype 4 | p-value |
| --- | --- | --- | --- | --- | --- |
| Hilar and mediastinal lymphadenopathy | 100,00% | 80,65% | 100,00% | 80,00% | **0,0017** |
| Lymph node calcifications | 6,78% | 12,90% | 35,71% | 20,00% | **0,0334** |
| Bronchovascular nodules | 22,03% | 67,74% | 100,00% | 20,00% | **<0,0001** |
| Parenchymal nodules | 30,51% | 100,00% | 100,00% | 80,00% | **<0,0001** |
| Subpleural nodules | 55,93% | 96,77% | 100,00% | 100,00% | **<0,0001** |
| Confluent nodules | 8,47% | 41,94% | 85,71% | 40,00% | **<0,0001** |
| Sharp-marginated nodules | 32,20% | 70,97% | 92,86% | 80,00% | **<0,0001** |
| Peripheral consolidation | 7,02% | 22,58% | 50,00% | 40,00% | **0,0012** |
| Central consolidations | 5,08% | 19,35% | 85,71% | 60,00% | **<0,0001** |
| Gound-glass | 30,51% | 32,26% | 35,71% | 40,00% | 0,9608 |
| Mosaic pattern | 5,08% | 3,23% | 0,00% | 0,00% | 0,7831 |
| Architectural distortion | 16,95% | 54,84% | 71,43% | 80,00% | **<0,0001** |
| Septal line, | 25,42% | 83,87% | 92,86% | 80,00% | **<0,0001** |
| Honeycombing | 3,39% | 19,35% | 42,86% | 0,00% | **0,0005** |
| Bronchial deformation | 8,47% | 61,29% | 78,57% | 80,00% | **<0,0001** |
| Bronchiectasis | 8,47% | 41,94% | 14,29% | 60,00% | **0,0003** |
| Pleural thickening | 16,95% | 41,94% | 50,00% | 60,00% | **0,0088** |
| Parenchymal calcifications | 1,69% | 3,23% | 7,14% | 20,00% | 0,1779 |
| Aspergiloma | 0,00% | 0,00% | 0,00% | 0,00% | - |

*Statistically significant correlations (p < 0.05) are marked in bold.
