# Supplementary material for: Expert intuition vs. Algorithmic clustering: Mapping radiological subtypes in pulmonary sarcoidosis
Source: PLoS One. 2026 Oct 15;21(10):e0358960. doi: 10.1371/journal.pone.0358960 (PMC13671569; doi:10.1371/journal.pone.0358960)
Supplement: Supplementary file 3 [file pone.0358960.s003.docx]

**Table S3. Frequency of individual radiological features observed in different groups**

|  | Group A | Group B | Group C | Group D | p-value |
| --- | --- | --- | --- | --- | --- |
| Hilar and mediastinal lymphadenopathy | 79,17% | 100,00% | 94,44% | 100,00% | **0,0099** |
| Lymph node calcifications | 0,00% | 3,57% | 27,78% | 15,79% | **0,0051** |
| Bronchovascular nodules | 54,17% | 0,00% | 88,89% | 10,53% | **<0,0001** |
| Parenchymal nodules, | 91,67% | 7,14% | 94,44% | 36,84% | **<0,0001** |
| Subpleural nodules | 87,50% | 17,86% | 97,22% | 100,00% | **<0,0001** |
| Confluent nodules | 8,33% | 3,57% | 69,44% | 15,79% | **<0,0001** |
| Sharp-marginated nodules | 62,50% | 0,00% | 88,89% | 57,89% | **<0,0001** |
| Peripheral consolidation | 0,00% | 3,57% | 50,00% | 5,26% | **<0,0001** |
| Central consolidations | 8,33% | 3,57% | 55,56% | 5,26% | **<0,0001** |
| Gound-glass | 33,33% | 35,71% | 22,22% | 47,37% | 0,2871 |
| Mosaic pattern | 4,17% | 3,57% | 0,00% | 10,53% | 0,2787 |
| Architectural distortion | 29,17% | 14,29% | 75,00% | 15,79% | **<0,0001** |
| Septal line | 50,00% | 17,86% | 91,67% | 36,84% | **<0,0001** |
| Honeycombing | 4,17% | 3,57% | 30,56% | 5,26% | **0,0022** |
| Bronchial deformation | 25,00% | 3,57% | 75,00% | 21,05% | **<0,0001** |
| Bronchiectasis | 25,00% | 3,57% | 41,67% | 5,26% | **0,0007** |
| Pleural thickening | 12,50% | 14,29% | 58,33% | 26,32% | **0,0002** |
| Parenchymal calcifications | 0,00% | 0,00% | 11,11% | 0,00% | **0,0421** |
| Aspergiloma | 0,00% | 0,00% | 0,00% | 0,00% | - |

*Statistically significant correlations (p < 0.05) are marked in bold.
