# Supplementary material for: Expert intuition vs. Algorithmic clustering: Mapping radiological subtypes in pulmonary sarcoidosis
Source: PLoS One. 2026 Oct 15;21(10):e0358960. doi: 10.1371/journal.pone.0358960 (PMC13671569; doi:10.1371/journal.pone.0358960)
Supplement: Supplementary file 4 [file pone.0358960.s004.docx]

**Table S4. One-way ANOVA models explaining the radiological subtype**

| **Parameter** | **p** | **eta^2** | **test** | **post-hoc** |
| --- | --- | --- | --- | --- |
| Van den Heuvel | **<0,0001** | **0,62** | HSD | 1 and 2, 3, 4 2 and 3 |
| FEV_1_ | **<0,0001** | **0,30** | HSD | 1 and 3, 4 2 and 4 |
| FVC | **0,0033** | 0,13 | HSD | no differences |
| TLCOc | **0,0053** | 0,12 | HSD | no differences |
| Abnormal spirometry | **0,24** | **0,15** | G-H | no differences |
| Abnormal dyfusion | **0,48** | **0,17** | G-H | no differences |

*Statistically significant correlations (p < 0.05) are marked in bold, eta^2>0,14

Van den Heuvel – result obtained in the Van den Heuvel questionnaire; FEV_1_ - forced expiratory volume in one second); FVC - forced vital capacity); TLCOc - lung transfer factor for carbon monoxide
